# Investigation of Type 2 Diabetes Risk Alleles Support *CDKN2A/B*, *CDKAL1*, and *TCF7L2* As Susceptibility Genes in a Han Chinese Cohort

**DOI:** 10.1371/journal.pone.0009153

**Published:** 2010-02-10

**Authors:** Jie Wen, Tina Rönn, Anders Olsson, Zhen Yang, Bin Lu, Yieping Du, Leif Groop, Charlotte Ling, Renming Hu

**Affiliations:** 1 Department of Endocrinology, Shanghai Medical College Fudan University, Shanghai, China; 2 Department of Clinical Sciences, Lund University Diabetes Center, Malmö, Sweden; Peninsula Medical School, United Kingdom

## Abstract

**Background:**

Recent genome-wide association studies (GWASs) have reported several genetic variants to be reproducibly associated with type 2 diabetes. Additional variants have also been detected from a metaanalysis of three GWASs, performed in populations of European ancestry. In the present study, we evaluated the influence of 17 genetic variants from 15 candidate loci, identified in type 2 diabetes GWASs and the metaanalysis, in a Han Chinese cohort.

**Methodology/Principal Findings:**

Selected type 2 diabetes–associated genetic variants were genotyped in 1,165 type 2 diabetic patients and 1,136 normoglycemic control individuals of Southern Han Chinese ancestry. The OR for risk of developing type 2 diabetes was calculated using a logistic regression model adjusted for age, sex, and BMI. Genotype-phenotype associations were tested using a multivariate linear regression model. Genetic variants in *CDKN2A/B*, *CDKAL1*, *TCF7L2*, *TCF2*, *MC4R*, and *PPARG* showed a nominal association with type 2 diabetes (*P*≤0.05), of whom the three first would stand correction for multiple testing: *CDKN2A/B* rs10811661, OR: 1.26 (1.12–1.43) *P* = 1.8*10^−4^; *CDKAL1* rs10946398, OR: 1.23 (1.09–1.39); *P* = 7.1*10^−4^, and *TCF7L2* rs7903146, OR: 1.61 (1.19–2.18) *P* = 2.3 * 10^−3^. Only nominal phenotype associations were observed, notably for rs8050136 in *FTO* and fasting plasma glucose (*P* = 0.002), postprandial plasma glucose (*P* = 0.002), and fasting C-peptide levels (*P* = 0.006) in the diabetic patients, and with BMI in controls (*P* = 0.033).

**Conclusions/Significance:**

We have identified significant association between variants in *CDKN2A/B*, *CDKAL1* and *TCF7L2*, and type 2 diabetes in a Han Chinese cohort, indicating these genes as strong candidates conferring susceptibility to type 2 diabetes across different ethnicities.

## Introduction

Type 2 diabetes is a complex polygenic disorder characterized by the presence of insulin resistance and pancreatic beta cell dysfunction. Interactions between environmental and genetic factors are involved in the onset and development of the disease. The prevalence of type 2 diabetes is increasing rapidly worldwide and China will be one of the countries hit hardest, with the diabetic population more than doubling in the next 20 years [1].

Many genetic variants have been associated with type 2 diabetes, but from a long list of candidate genes only three have unambiguously been associated with the disease: *PPARG*, *KCNJ11* and *TCF7L2* [2]–[4]. However, in 2007, several reproducible genome-wide association studies (GWASs) confirmed these well-established susceptibility genes and identified a number of new loci (*SLC30A8*, *HHEX, CDKN2A/B*, *IGF2BP2*, *GCKR*, *FTO*, and *CDKAL1*) at which common variants influence risk of type 2 diabetes in Europeans [5]–[10].

Intriguingly, another study in 2007 showed that a variant in *TCF2* was associated with increased risk of prostate cancer but reduced risk of type 2 diabetes in individuals of European, African and Asian descent [11]. Furthermore, a meta-analysis of three GWASs detected six novel variants (in *JAZF1*, *CDC123/CAMK1D*, *TSPAN8/LGR5*, *THADA*, *ADAMTS9*, and *NOTCH2*) that were associated with type 2 diabetes [12]. Recently, two GWASs established that a common genetic variant near *MC4R* gene (rs17782313) was associated with increased obesity risk and insulin resistance [13], [14].

Most of the genes associated with type 2 diabetes (*TCF7L2*, *SLC30A8*, *HHEX*, *CDKAL1*, *CDKN2A/B* and *IGF2BP2*) might be implicated in beta cell function [8]–[10], [15]–[17]. In addition, variation in *GCKR*, encoding glucokinase regulatory protein, and *FTO*, the fat mass and obesity associated gene, were associated with serum triglyceride and BMI respectively [5], [6].

Most of the populations analyzed in the GWASs were of European ancestry and the contributions of these genetic variants in other ethnic groups are less clear. Nevertheless, some variants associated with risk of type 2 diabetes identified by GWASs in Europeans have been replicated in Asians. However, due to the ethnic differences in risk allele frequencies, the impact of these genes varies between these two ethnic groups [18]–[21]. Although studies have failed to show association between the previously reported risk allele rs7903146 in *TCF7L2* with type 2 diabetes in Chinese, it has been suggested that variations in this gene confer risk of type 2 diabetes in this ethnic group. Interestingly, two other *TCF7L2* SNPs (rs11196218 and rs290487) were found to associate with type 2 diabetes in Chinese [22]–[24]. Moreover, no study has so far examined if the variants identified in the meta-analysis are associated with type 2 diabetes in a Chinese population.

To obtain a global view of the role of these SNPs in the pathogenesis of type 2 diabetes worldwide, it is important to test associations between candidate SNPs and type 2 diabetes in various ethnic groups. In the present study we therefore evaluated the influence of 17 type 2 diabetes associated SNPs in 15 candidate loci in a Han Chinese population. As some variants are known to affect the risk of type 2 diabetes through obesity, and others have shown the strongest association with related metabolic traits, we also investigated the genetic impact on BMI, glucose levels, C-peptide, and triglycerides.

## Materials and Methods

### Participants

All studied individuals were of Southern Han Chinese ancestry residing in the Shanghai metropolitan area. 1165 type 2 diabetic patients were recruited from the Endocrinology and Metabolism outpatient clinics at Fudan University Huashan Hospital in Shanghai, China. Type 2 diabetes mellitus was diagnosed according to 1999 WHO criteria [25]. All diabetic patients were unrelated and diagnosed after the age of 27 years. Known subtypes of diabetes were excluded based on antibody measurements and inheritance. The 1136 non-diabetic unrelated control individuals were older than 45 years, had no family history of diabetes mellitus and normal glucose tolerance was verified by an OGTT. The clinical characteristics of participants are summarized in Table 1. Measurement of C-peptide was only obtained for the diabetics. Written informed consent was obtained from all participants and the study was approved by the Ethics Committee of Huashan Hospital affiliated to Fudan University.

**Table 1:** Clinical characteristics of the participants.

	Type 2 diabetes cases	Controls
*N* (male/female)	1165 (455/710)	1136 (353/783)
Age (years)	60.3±10.9	59.1±7.9
BMI (kg/m^2^)	25.2±3.4	24.1±3.0
Fasting C-peptide (nmol/l)	1.09 (0.62)	n/a
Fasting plasma glucose (mmol/l)	8.4±3.0	5.2±0.4
2 h postprandial plasma glucose (mmol/l)	15.1±5.3	6.0±1.0
Triglycerides (mmol/l)	1.65 (1.15)	1.23 (0.87)

Data are expressed as mean ± SD for normally distributed values (age, BMI and glucose) and median (IQR) for non-normally distributed values (C-peptide and triglycerides).

### Genotyping

Genomic DNA was extracted from peripheral blood leukocytes using the conventional phenol/chloroform method. SNP selection was based on published type 2 diabetes GWAS data and a meta-analysis of those, as summarized in the introduction. SNPs in *NOTCH2*, *THADA* and *WFS1* were not included as they have a MAF <0.05 in Chinese as reported by the HapMap project, which would limit the power to detect an association. Two of the *TCF7L2* polymorphisms (rs290487 and rs7903146) were genotyped using TaqMan allelic discrimination assays (Applied Biosystems, Foster City, CA, USA). All other SNPs were genotyped using iPLEX (Sequenom, San Diego, CA, USA) and detected by matrix-assisted laser desorption/ionisation-time of flight mass spectrometry. All analyzed SNPs are presented in Table 2, except rs13266634 (*SLC30A8*), which failed genotyping although applying two different methods. The genotype frequencies were all in Hardy-Weinberg equilibrium (*P*>0.05) and 96 samples (4%) were run in duplicates with a 100% concordance rate.

**Table 2:** Genotypic and allelic distribution of type 2 diabetes susceptibility SNPs and association with type 2 diabetes in a Han Chinese cohort.

Nearest gene(s)	SNP	Alleles^a^ major/minor	MAF	Genotype Frequency T2D Cases Controls	OR_add_ (95% CI)^b^	*P* value^c^	Power^d^
*CDKN2A/B*	rs10811661	**T**/C	0.475	0.351/0.457/0.1920.271/0.510/0.220	1.26 (1.12–1.43)	1.8*10^−4^	91%
*CDKAL1*	rs10946398	A/**C**	0.392	0.319/0.481/0.2000.369/0.478/0.153	1.23 (1.09–1.39)	7.1*10^−4^	87%
*TCF7L2*	rs7903146	C/**T**	0.033	0.897/0.103/0.0000.938/0.060/0.003	1.61 (1.19–2.18)	2.3*10^−3^	59%
*TCF2*	rs4430796	T/**C**	0.293	0.456/0.442/0.1020.498/0.419/0.083	1.16 (1.02–1.32)	0.026	35%
*MC4R*	rs17782313	T/**C**	0.191	0.610/0.340/0.0500.652/0.312/0.035	1.18 (1.01–1.37)	0.032	13%
*PPARG*	rs1801282	**C**/G	0.064	0.901/0.099/0.0000.877/0.118/0.005	1.30 (1.00–1.68)	0.050	46%
*JAZF1*	rs864745	**A**/G	0.225	0.643/0.316/0.0410.608/0.334/0.058	1.16 (1.00–1.34)	0.054	29%
*HHEX/IDE*	rs1111875	T/**C**	0.279	0.485/0.423/0.0920.517/0.408/0.075	1.14 (1.00–1.30)	0.056	52%
*GCKR*	rs780094	**T**/C	0.481	0.283/0.519/0.1970.260/0.520/0.221	1.11 (0.98–1.26)	0.088	5%
*IGF2BP2*	rs4402960	C/**A**	0.253	0.541/0.378/0.0810.565/0.364/0.071	1.12 (0.98–1.28)	0.11	71%
*FTO*	rs8050136	C/**A**	0.119	0.748/0.236/0.0160.774/0.216/0.011	1.15 (0.96–1.38)	0.14	47%
*KCNJ11*	rs5219	G/**A**	0.398	0.339/0.504/0.1570.374/0.455/0.170	1.07 (0.95–1.21)	0.26	96%
*TCF7L2*	rs11196218	G/**A**	0.262	0.509/0.444/0.0500.549/0.378/0.073	1.07 (0.93–1.23)	0.34	100%
*TSPAN8/LGR5*	rs7961581	T/**C**	0.202	0.636/0.311/0.0530.638/0.321/0.041	1.05 (0.90–1.22)	0.55	24%
*TCF7L2*	rs290487	T/**C**	0.362	0.404/0.462/0.1340.409/0.461/0.131	1.00 (0.88–1.13)	0.99	100%
*CDC123/CAMK1D*	rs12779790	A/**G**	0.164	0.697/0.268/0.0360.699/0.276/0.026	1.02 (0.87–1.20)	0.80	29%
*ADAMTS9*	rs4607103	**C**/T	0.369	0.408/0.446/0.1450.406/0.450/0.144	1.00 (0.89–1.13)	0.96	32%

a Risk allele denoted in bold.

b Calculated using logistic regression, assuming an additive model adjusted for age, sex and BMI.

c *P*-values shown are not corrected for multiple testing.

d Assuming an additive model, a T2D frequency of 6%, α = 0.05, MAF based on this study and OR as previously reported.

MAF, minor allele frequency in control samples; T2D, type 2 diabetes.

### Statistical Analyses

The OR for risk of developing type 2 diabetes was calculated using logistic regression, assuming an additive genetic model, adjusted for age (age of diagnosis for cases and age at participation for controls), sex and BMI. Power to detect an association was calculated for each SNP using the Genetic Power Calculator [26], assuming an additive model, a type 2 diabetes frequency of 6%, using MAF as observed in the studied cohort, α = 0.05, and effect size (OR) as previously reported [2], [3], [5], [11], [12], [22], [23] (Table 2). Multivariate linear regression analyses were used to test genotype-phenotype correlations and adjusted for age, sex and BMI (apart from the BMI phenotype). Non-normally distributed values were log-transformed before analysis. All statistical analyses were performed using either SPSS program version 14.0 for Windows (SPSS, Chicago, IL, USA) or NCSS software, 2004 release (NCSS, Kaysville, UT, USA).

## Results

The clinical characteristics of participating individuals are presented in Table 1.

### Type 2 Diabetes Susceptibility SNPs and Association with the Disease in Han Chinese

17 SNPs were analyzed for association with type 2 diabetes in the studied Han Chinese individuals. Genotype and allele frequencies are shown in Table 2 together with results of the association analyses. Power to detect an association based on here observed MAF and OR as reported previously varied from 5–100%, with only five SNPs having more than 80% power (Table 2). SNPs in *CDKN2A/B*, *CDKAL1*, *TCF7L2*, *TCF2*, *MC4R* and *PPARG* showed a nominal association with type 2 diabetes (*P*≤0.05), of whom the three first would stand correction for multiple testing: rs10811661, OR: 1.26 (1.12–1.43) *P* = 1.8*10^−4^; rs10946398, OR: 1.23 (1.09–1.39); *P* = 7.1*10^−4^ and rs7903146, OR: 1.61 (1.19–2.18) *P* = 2.3*10^−3^ (Table 2). As both *FTO* and *MC4R* are known to affect type 2 diabetes risk through modulation of obesity, association was also calculated without adjustment for BMI. Both variants showed a modest increase in OR and a slightly lower *P*-value (rs8050136, OR: 1.18 (0.99–1.41) *P* = 0.066 and rs17782313, OR: 1.20 (1.04–1.39); *P* = 0.015).

### Association of 17 Genetic Variants Related to Type 2 Diabetes and Metabolic Quantitative Traits

We examined associations between all the analyzed SNPs and metabolic quantitative traits in cases, controls and also in cases and controls combined (except for the glucose phenotypes; Table S1). The metabolic phenotypes tested include BMI, fasting plasma glucose, 2 h postprandial plasma glucose, C-peptide (only for cases) and triglycerides. No association was observed after correction for multiple testing, although, the *A* allele of rs8050136 (*FTO*) showed nominal associations with fasting plasma glucose (*P* = 0.002), postprandial plasma glucose (*P* = 0.002) and the fasting C-peptide levels (*P* = 0.006) in the cases. There was no association between this SNP and BMI in the diabetic cases, but an association was found between the *FTO* SNP and BMI in the non-diabetic controls and when combining all individuals (*P* = 0.033 and 0.031 respectively). Additionally, the risk C allele of rs10946398 (*CDKAL1*) suggest an increase in fasting plasma glucose in normoglycemic controls (*P* = 0.016) and a nominal association was also observed between the A allele of rs11196218 (*TCF7L2*) and a decrease in C-peptide in the cases (Table S1).

## Discussion

In the present study, we analyzed 17 SNPs in a type 2 diabetes case-control cohort comprising 2301 Han Chinese individuals. The majority of the investigated SNPs have previously been identified conferring risk of type 2 diabetes, but these studies were mainly performed in Europeans. We replicated previous findings of associations for three SNPs in this Chinese population (rs10811661 in *CDKN2A/B*, rs10946398 in *CDKAL1*, and rs7903146 in *TCF7L2*) suggesting that some of the variants associated with type 2 diabetes in Europeans are also associated with the disease in Asians. In addition, we have previously reported an association for *MTNR1B* and type 2 diabetes in this cohort [21].

GWASs have recently described novel type 2 diabetes susceptibility loci, including several previously unknown genomic regions, such as *CDKN2A/B* and *CDKAL1* [5], [7]–[10]. We observed a significant association between *CDKN2A/B* rs10811661 and type 2 diabetes (OR: 1.26, *P* = 1.8*10^−4^) in Chinese. The OR in our study is similar to the one reported in Europeans (OR: 1.20) [5], [7], [10]. However, the risk allele (T) is less prevalent in Chinese Hans (risk allele frequency  = 0.52) compared with Europeans (risk allele frequency  = 0.83) [5]. We also replicated the diabetes susceptibility variant rs10946398 in *CDKAL1* (OR: 1.2, *P* = 7.1*10^−4^). Our results support previous findings that these variants in *CDKN2A/B* and *CDKAL1* individually contribute to the risk of type 2 diabetes in the Han Chinese population [20], [22], but imply some ethnic differences between Europeans and Asians.

The *TCF7L2* polymorphism rs7903146 is the strongest single genetic variant associated with type 2 diabetes [4], [5], [7], [8], [10] and has been convincingly replicated in multiple populations [27]–[30]. In contrast to populations of European and African ancestries, the risk T-allele of rs7903146 was rare in our studied Chinese cohort with a MAF of 3.3%. This is in concordance with the data reported by the HapMap project [31]. However, in contrast to earlier studies performed in Chinese cohorts [22]–[24], we found a significant association between rs7903146 and type 2 diabetes (OR = 1.61, *P* = 2.3*10^−3^). The inability to detect this association in the previous Chinese studies may be due to insufficient power. Notably, we could not replicate two other susceptibility SNPs in *TCF7L2* (rs11196218 and rs290487), previously reported to be associated with type 2 diabetes in Chinese studies [22], [23]. However, we did identify a consistent association of this gene with type 2 diabetes in our Chinese population, further validating the contribution of *TCF7L2* on susceptibility to the disease. Since the risk allele frequency of rs7903146 is lower in Chinese compared with i.e. Europeans, the genetic contribution of this polymorphism to type 2 diabetes on a population level is relatively small. Interestingly, two recent case-control studies independently reported significant associations between rs7903146 and type 2 diabetes in the Japanese [32], [33], supporting our result that rs7903146 may contribute to diabetes susceptibility in East Asian populations.

None of the other investigated SNPs showed significant association with type 2 diabetes in our cohort. This may be explained by different environmental risk profiles between Europeans and Asians, body composition and genetic backgrounds, or that we have insufficient power with current sample size to replicate some of these previously reported risk variants. Moreover, our study is the first to investigate the SNPs identified by a meta-analysis of three GWASs [12] in a Chinese case-control cohort. However, it is not unexpected that our study was unable to find significant associations between SNPs in *JAZF1*, *CDC123/CAMK1D*, *TSPAN8/LGR5* and *ADAMTS9* and type 2 diabetes, since the meta-analysis required more than 9000 samples for an 80% power [12].

Of all the analyzed SNPs, *FTO* showed the strongest association with metabolic traits. There was a trend towards elevated levels of fasting plasma glucose and 2 h postprandial glucose in the diabetic *A*-allele carriers, as well as a decreased level of fasting C-peptide in the same group. In this study, we also confirmed a nominal association between rs8050136 and BMI in non-diabetic controls. The association between *FTO* and obesity has been shown to indirectly modulate risk of type 2 diabetes in Europeans [6], [10], [34], but it has been difficult to demonstrate an association between *FTO* (rs8050136) and obesity or BMI in Asians [35]–[37]. Nevertheless, our result, together with data from Ng et al. [19], indicates that *FTO* also affects BMI in Asians.

In summary, we have identified significant associations between variants in *CDKN2A/B*, *CDKAL1* and *TCF7L2* and type 2 diabetes in a Han Chinese population. Our results indicate that these genes are strong candidates conferring susceptibility to type 2 diabetes across different ethnicities. However, more comprehensive studies in larger populations of different ethnic backgrounds are needed to clarify the molecular mechanisms and underlying genetic architecture of type 2 diabetes.

## Supporting Information

Table S1 Effect of studied genetic variants on metabolic quantitative traits in type 2 diabetic cases and normoglycemic controls.
(0.28 MB DOC)
