# Supplementary material for: Investigation of Type 2 Diabetes Risk Alleles Support CDKN2A/B, CDKAL1, and TCF7L2 As Susceptibility Genes in a Han Chinese Cohort
Source: PLoS One. 2010 Feb 10;5(2):e9153. doi: 10.1371/journal.pone.0009153 (PMC2818850; doi:10.1371/journal.pone.0009153)
Supplement: Table S1 — Effect of studied genetic variants on metabolic quantitative traits in type 2 diabetic cases and normoglycemic controls. (0.28 MB DOC) [file pone.0009153.s001.doc]

**Table S1 Effect of studied genetic variants on metabolic quantitative traits in type 2 diabetic cases and normoglycemic controls**

| SNP ID Nearest gene | Genotype | BMI (kg/m^2^) | | | FPG (mmol/l) | | PPG (mmol/l) | | TG (mmol/l) | | | CP_0_ (nmol/l) |
| --- | --- | --- | --- | --- | --- | --- | --- | --- | --- | --- | --- | --- |
|  |  | Case | Control | All | Case | Control | Case | Control | Case | Control | All | Case |
| rs10811661  *CDKN2A/B* | TT | 25.14 ± 3.27 | 24.02 ± 3.32 | 24.65 ± 3.34 | 8.36 ± 2.66 | 5.20 ± 0.39 | 15.21 ± 5.14 | 5.93 ± 1.08 | 1.70 (1.19) | 1.19 (0.88) | 1.47 (1.13) | 1.08 (0.66) |
|  | TC | 25.22 ± 3.47 | 24.15 ± 2.92 | 24.65 ± 3.24 | 8.44 ± 3.28 | 5.22 ± 0.39 | 15.07 ± 5.62 | 6.04 ± 1.03 | 1.63 (1.15) | 1.21 (0.87) | 1.44 (1.03) | 1.08 (0.56) |
|  | CC | 25.78 ± 3.64 | 24.11 ± 3.01 | 24.88 ± 3.42 | 8.19 ± 2.75 | 5.20 ± 0.35 | 14.74 ± 4.96 | 6.09 ± 0.97 | 1.63 (1.23) | 1.30 (0.85) | 1.42 (1.05) | 1.21 (0.58) |
| Beta (sem) ^a^ | | 0.28 (0.15) | 0.05 (0.13) | 0.11 (0.10) | -0.051 (0.13) | -0.001 (0.016) | -0.16 (0.23) | 0.074 (0.044) | -0.004 (0.023) | 0.026 (0.022) | -0.003 (0.016) | 0.016 (0.026) |
| *P* value^b^ | | 0.052 | 0.69 | 0.29 | 0.69 | 0.94 | 0.47 | 0.098 | 0.86 | 0.24 | 0.84 | 0.54 |
| rs10946398  *CDKAL1* | AA | 25.46 ± 3.43 | 24.16 ± 2.89 | 24.77 ± 3.21 | 8.28 ± 2.86 | 5.19 ± 0.37 | 14.85 ± 5.12 | 6.02 ± 1.03 | 1.72 (1.27) | 1.19 (0.80) | 1.45 (1.12) | 1.15 (0.65) |
|  | AC | 25.20 ± 3.46 | 24.07 ± 3.16 | 24.64 ± 3.36 | 8.39 ± 3.13 | 5.20 ± 0.37 | 14.99 ± 5.36 | 5.98 ± 1.05 | 1.61 (1.09) | 1.25 (0.90) | 1.43 (1.01) | 1.08 (0.65) |
|  | CC | 25.07 ± 3.33 | 24.09 ± 2.87 | 24.65 ± 3.17 | 8.34 ± 2.81 | 5.28 ± 0.39 | 15.08 ± 5.45 | 6.18 ± 0.98 | 1.63 (1.23) | 1.22 (0.88) | 1.44 (1.12) | 1.06 (0.55) |
| Beta (sem) ^a^ | | -0.22 (0.14) | -0.06 (0.13) | -0.07 (0.10) | 0.007 (0.13) | 0.039 (0.016) | 0.12 (0.22) | 0.059 (0.044) | -0.040 (0.023) | 0.027 (0.022) | 0.006 (0.016) | -0.047 (0.025) |
| *P* value^b^ | | 0.13 | 0.67 | 0.45 | 0.96 | 0.016 | 0.60 | 0.18 | 0.079 | 0.21 | 0.69 | 0.065 |
| rs7903146  *TCF7L2* | CC | 25.21 ± 3.36 | 24.17 ± 3.05 | 24.68 ± 3.25 | 8.32 ± 2.92 | 5.22 ± 0.38 | 14.99 ± 5.29 | 6.04 ± 1.04 | 1.64 (1.16) | 1.23 (0.87) | 1.44 (1.05) | 1.10 (0.60) |
|  | CT | 25.55 ± 3.91 | 23.65 ± 3.04 | 24.86 ± 3.72 | 8.99 ± 3.86 | 5.17 ± 0.39 | 15.80 ± 5.78 | 5.94 ± 1.00 | 1.87 (1.29) | 1.34 (0.72) | 1.56 (1.16) | 1.08 (0.69) |
|  | TT |  | 22.23 ± 2.20 | 22.23 ± 2.20 |  | 5.43 ± 0.42 |  | 5.77 ± 1.55 |  | 1.23 (1.33) | 1.23 (1.33) |  |
| Beta (sem) ^a^ | | 0.38 (0.33) | -0.60 (0.35) | 0.08 (0.25) | 0.70 (0.29) | -0.017 (0.043) | 0.82 (0.52) | -0.069 (0.12) | 0.012 (0.052) | 0.004 (0.058) | 0.028 (0.040) | -0.007 (0.057) |
| *P* value^b^ | | 0.26 | 0.091 | 0.73 | 0.017 | 0.70 | 0.11 | 0.56 | 0.81 | 0.94 | 0.48 | 0.90 |
| rs4430796  *TCF2* | TT | 25.23 ± 3.54 | 24.15 ± 3.11 | 24.67 ± 3.37 | 8.40 ± 3.04 | 5.23 ± 0.38 | 15.24 ± 5.37 | 6.07 ± 1.00 | 1.73 (1.26) | 1.23 (0.77) | 1.45 (1.02) | 1.10 (0.59) |
|  | TC | 25.38 ± 3.32 | 24.09 ± 3.03 | 24.76 ± 3.25 | 8.32 ± 2.93 | 5.20 ± 0.38 | 14.91 ± 5.29 | 5.99 ± 1.06 | 1.62 (1.16) | 1.21 (0.91) | 1.44 (1.08) | 1.09 (0.60) |
|  | CC | 24.65 ± 3.22 | 24.38 ± 2.82 | 24.53 ± 3.04 | 8.66 ± 3.38 | 5.23 ± 0.38 | 15.01 ± 5.55 | 5.98 ± 1.10 | 1.63 (1.06) | 1.27 (1.16) | 1.51 (1.22) | 1.06 (0.73) |
| Beta (sem) ^a^ | | -0.13 (0.15) | 0.05 (0.14) | -0.01 (0.11) | 0.042 (0.14) | -0.014 (0.017) | -0.21 (0.24) | -0.062 (0.048) | -0.034 (0.024) | 0.022 (0.023) | 0.000 (0.017) | -0.009 (0.028) |
| *P* value^b^ | | 0.41 | 0.75 | 0.92 | 0.76 | 0.41 | 0.39 | 0.19 | 0.16 | 0.36 | 0.98 | 0.74 |
| rs17782313  *MC4R* | TT | 25.20 ± 3.44 | 24.05 ± 3.02 | 24.62 ± 3.28 | 8.47 ± 3.08 | 5.21 ± 0.38 | 15.13 ± 5.26 | 6.04 ± 1.03 | 1.68 (1.21) | 1.23 (0.86) | 1.46 (1.07) | 1.10 (0.57) |
|  | TC | 25.27 ± 3.32 | 24.23 ± 3.01 | 24.78 ± 3.22 | 8.24 ± 3.00 | 5.21 ± 0.37 | 14.92 ± 5.63 | 5.97 ± 1.06 | 1.62 (1.19) | 1.27 (0.83) | 1.44 (1.09) | 1.09 (0.69) |
|  | CC | 25.87 ± 3.91 | 24.18 ± 2.83 | 25.17 ± 3.59 | 8.07 ± 2.41 | 5.26 ± 0.41 | 14.43 ± 4.31 | 6.05 ± 1.04 | 1.44 (0.83) | 1.22 (1.03) | 1.36 (1.02) | 1.02 (0.58) |
| Beta (sem) ^a^ | | 0.16 (0.17) | 0.14 (0.16) | 0.21 (0.12) | -0.22 (0.15) | 0.002 (0.020) | -0.21 (0.27) | -0.050 (0.055) | -0.054 (0.027) | 0.018 (0.027) | -0.009 (0.020) | -0.019 (0.032) |
| *P* value^b^ | | 0.36 | 0.40 | 0.082 | 0.15 | 0.91 | 0.43 | 0.37 | 0.049 | 0.50 | 0.65 | 0.56 |
| rs1801282  *PPARG* | CC | 25.26 ± 3.41 | 24.12 ± 3.02 | 24.70 ± 3.27 | 8.42 ± 3.00 | 5.21 ± 0.38 | 15.11 ± 5.31 | 6.03 ± 1.03 | 1.65 (1.17) | 1.23 (0.87) | 1.45 (1.07) | 1.09 (0.62) |
|  | CG | 25.02 ± 3.46 | 24.12 ± 3.25 | 24.53± 3.37 | 7.93 ± 2.79 | 5.22 ± 0.37 | 14.41 ± 5.36 | 6.06 ± 1.06 | 1.62 (1.01) | 1.22 (0.85) | 1.39 (0.94) | 1.16 (0.53) |
|  | GG | . | 23.98 ± 2.33 | 23.98 ± 2.33 |  | 5.20 ± 0.20 |  | 5.53 ± 1.22 |  | 1.08 (0.95) | 1.08 (0.95) |  |
| Beta (sem) ^a^ | | -0.27 (0.34) | -0.01 (0.26) | -0.19 (0.21) | -0.51 (0.30) | 0.005 (0.032) | -0.72 (0.53) | -0.018 (0.086) | -0.025 (0.053) | -0.011(0.042) | -0.030 (0.034) | -0.010 (0.061) |
| *P* value^b^ | | 0.42 | 0.96 | 0.38 | 0.082 | 0.88 | 0.17 | 0.84 | 0.64 | 0.79 | 0.39 | 0.87 |
| rs864745  *JAZF1* | AA | 25.19 ± 3.49 | 24.14 ± 3.15 | 24.68 ± 3.37 | 8.27 ± 2.93 | 5.22 ± 0.38 | 14.76 ± 5.27 | 6.07 ± 1.02 | 1.68 (1.20) | 1.21 (0.83) | 1.45 (1.13) | 1.11 (0.62) |
|  | AG | 25.27 ± 3.34 | 24.09 ± 2.86 | 24.67 ± 3.16 | 8.57 ± 3.12 | 5.22 ± 0.37 | 15.48 ± 5.44 | 5.96 ± 1.02 | 1.62 (1.13) | 1.26 (0.93) | 1.44 (1.01) | 1.08 (0.60) |
|  | GG | 25.11 ± 3.19 | 24.43 ± 3.01 | 24.71 ± 3.09 | 8.18 ± 3.39 | 5.18 ± 0.39 | 14.79 ± 5.15 | 5.98 ± 1.16 | 1.38 (1.08) | 1.25 (0.96) | 1.33 (1.03) | 1.16 (0.48) |
| Beta (sem) ^a^ | | 0.01 (0.18) | 0.05 (0.15) | 0.00 (0.12) | 0.20 (0.16) | -0.010 (0.019) | 0.46 (0.28) | -0.083 (0.051) | -0.018 (0.029) | 0.006 (0.025) | -0.014 (0.020) | -0.007 (0.033) |
| *P* value^b^ | | 0.94 | 0.73 | 1.00 | 0.22 | 0.61 | 0.10 | 0.10 | 0.54 | 0.81 | 0.49 | 0.84 |
| rs1111875  *HHEX/IDE* | TT | 25.31 ± 3.52 | 24.21 ± 3.13 | 24.73 ± 3.37 | 8.47 ± 3.27 | 5.21 ± 0.39 | 15.13 ± 5.34 | 6.02 ± 1.01 | 1.68 (1.22) | 1.20 (0.89) | 1.42 (1.17) | 1.10 (0.59) |
|  | TC | 25.34 ± 3.34 | 24.10 ± 3.00 | 24.72 ± 3.23 | 8.36 ± 2.78 | 5.21 ± 0.36 | 14.97 ± 5.25 | 6.02 ± 1.07 | 1.64 (1.24) | 1.27 (0.86) | 1.47 (1.00) | 1.14 (0.66) |
|  | CC | 24.76 ± 3.18 | 23.95 ± 2.92 | 24.40 ± 3.08 | 7.91 ± 2.43 | 5.22 ± 0.38 | 14.87 ± 5.18 | 6.12 ± 1.04 | 1.59 (1.12) | 1.17 (0.75) | 1.40 (1.02) | 1.00 (0.47) |
| Beta (sem) ^a^ | | -0.13 (0.16) | -0.12 (0.15) | -0.11 (0.11) | -0.22 (0.14) | 0.006 (0.018) | -0.19 (0.25) | 0.033 (0.049) | -0.019 (0.025) | 0.030 (0.024) | 0.010 (0.018) | -0.021 (0.029) |
| *P* value^b^ | | 0.43 | 0.42 | 0.34 | 0.12 | 0.74 | 0.45 | 0.51 | 0.45 | 0.21 | 0.59 | 0.46 |
| rs780094  *GCKR* | TT | 25.46 ± 3.52 | 24.17 ± 2.98 | 24.85 ± 3.37 | 8.14 ± 2.88 | 5.20 ± 0.38 | 15.05 ± 5.33 | 6.02 ± 0.99 | 1.70 (1.18) | 1.25 (0.82) | 1.46 (1.09) | 1.11 (0.62) |
|  | TC | 25.12 ± 3.38 | 24.11 ± 2.99 | 24.62 ± 3.23 | 8.54 ± 3.07 | 5.24 ± 0.38 | 15.22 ± 5.49 | 6.03 ± 1.06 | 1.66 (1.26) | 1.25 (0.91) | 1.47 (1.12) | 1.08 (0.63) |
|  | CC | 25.18 ± 3.35 | 24.19 ± 3.27 | 24.66 ± 3.34 | 8.35 ± 3.11 | 5.19 ± 0.36 | 14.66 ± 4.92 | 6.02 ± 1.05 | 1.61 (0.99) | 1.16 (0.86) | 1.36 (0.98) | 1.14 (0.61) |
| Beta (sem) ^a^ | | -0.15 (0.15) | -0.01 (0.13) | -0.11 (0.10) | 0.11 (0.13) | -0.001 (0.016) | -0.20 (0.23) | 0.003 (0.044) | -0.035 (0.023) | -0.015(0.021) | -0.029 (0.016) | -0.002 (0.027) |
| *P* value^b^ | | 0.31 | 0.93 | 0.29 | 0.39 | 0.94 | 0.39 | 0.95 | 0.13 | 0.49 | 0.073 | 0.94 |
| rs4402960  *IGF2BP2* | CC | 25.21 ± 3.39 | 24.26 ± 3.07 | 24.73 ± 3.27 | 8.42 ± 3.03 | 5.21 ± 0.37 | 15.09 ± 5.11 | 6.00 ± 1.03 | 1.68 (1.26) | 1.23 (0.88) | 1.46 (1.13) | 1.14 (0.60) |
|  | CA | 25.41 ± 3.50 | 24.01 ± 3.04 | 24.73 ± 3.56 | 8.25 ± 2.97 | 5.22 ± 0.39 | 14.88 ± 5.55 | 6.04 ± 1.07 | 1.62 (1.09) | 1.23 (0.91) | 1.42 (1.01) | 1.07 (0.60) |
|  | AA | 25.09 ± 3.33 | 23.57 ± 2.61 | 24.40 ± 3.11 | 8.58 ± 3.00 | 5.20 ± 0.37 | 15.02 ± 5.56 | 6.11 ± 0.98 | 1.61 (1.26) | 1.12 (0.78) | 1.37 (1.03) | 0.97 (0.63) |
| Beta (sem) ^a^ | | 0.04 (0.16) | -0.29 (0.15) | -0.09 (0.11) | -0.036 (0.14) | 0.003 (0.018) | -0.059 (0.25) | 0.072 (0.049) | -0.035 (0.025) | -0.015 (0.024) | -0.020 (0.018) | -0.025 (0.028) |
| *P* value^b^ | | 0.82 | 0.045 | 0.41 | 0.80 | 0.88 | 0.81 | 0.14 | 0.17 | 0.53 | 0.27 | 0.37 |
| rs8050136  *FTO* | CC | 25.20 ± 3.30 | 24.04 ± 3.07 | 24.61 ± 3.24 | 8.24 ± 2.84 | 5.22 ± 0.38 | 14.81 ± 5.32 | 5.97 ± 1.06 | 1.65 (1.21) | 1.21 (0.90) | 1.43 (1.10) | 1.12 (0.65) |
|  | CA | 25.28 ± 3.77 | 24.37 ± 2.95 | 24.85 ± 3.44 | 8.65 ± 3.42 | 5.22 ± 0.38 | 15.51 ± 5.25 | 6.23 ± 0.94 | 1.63 (0.98) | 1.29 (0.69) | 1.48 (0.97) | 1.05 (0.52) |
|  | AA | 25.87 ± 3.62 | 25.78 ± 3.17 | 25.84 ± 3.39 | 10.56 ± 3.76 | 5.04 ± 0.31 | 18.69 ± 5.27 | 5.64 ± 1.20 | 2.31 (1.61) | 1.41 (0.96) | 1.97 (1.53) | 1.08 (0.75) |
| Beta (sem) ^a^ | | 0.11 (0.21) | 0.43 (0.20) | 0.32 (0.15) | 0.58 (0.19) | -0.025 (0.025) | 1.02 (0.33) | 0.16 (0.068) | 0.030 (0.033) | 0.024 (0.033) | 0.039 (0.024) | -0.11 (0.038) |
| *P* value^b^ | | 0.60 | 0.033 | 0.031 | 0.002 | 0.31 | 0.002 | 0.025 | 0.37 | 0.47 | 0.11 | 0.006 |
| rs5219  *KCNJ11* | GG | 25.57 ± 3.45 | 24.30 ± 2.90 | 24.91 ± 3.24 | 8.32 ± 3.07 | 5.25 ± 0.37 | 14.78 ± 5.32 | 6.03 ± 1.04 | 1.68 (1.23) | 1.26 (0.86) | 1.45 (1.07) | 1.13 (0.61) |
|  | GA | 25.07 ± 3.35 | 23.97 ± 3.06 | 24.55 ± 3.26 | 8.45 ± 2.98 | 5.19 ± 0.37 | 15.29 ± 5.38 | 6.02 ± 1.04 | 1.67 (1.20) | 1.21 (0.90) | 1.48 (1.14) | 1.09 (0.61) |
|  | AA | 25.14 ± 3.55 | 24.13 ± 3.26 | 24.62 ± 3.44 | 8.30 ± 3.14 | 5.20 ± 0.39 | 14.83 ± 5.21 | 6.07 ± 0.99 | 1.54 (0.92) | 1.23 (0.91) | 1.38 (0.98) | 1.10 (0.71) |
| Beta (sem) ^a^ | | -0.26 (0.15) | -0.13 (0.13) | -0.19 (0.10) | 0.013 (0.13) | -0.030 (0.016) | 0.094 (0.23) | 0.024 (0.043) | -0.027 (0.023) | -0.011 (0.021) | -0.015 (0.016) | -0.009 (0.027) |
| *P* value^b^ | | 0.081 | 0.30 | 0.063 | 0.92 | 0.056 | 0.69 | 0.57 | 0.26 | 0.59 | 0.35 | 0.73 |
| rs11196218  *TCF7L2* | GG | 25.29 ± 3.53 | 24.17 ± 3.04 | 24.71 ± 3.34 | 8.37 ± 2.99 | 5.22 ± 0.39 | 14.89 ± 5.37 | 6.02 ± 1.04 | 1.69 (1.20) | 1.22 (0.78) | 1.43 (1.04) | 1.14 (0.62) |
|  | GA | 25.19 ± 3.29 | 24.08 ± 2.93 | 24.69 ± 3.18 | 8.32 ± 2.89 | 5.21 ± 0.37 | 15.17 ± 5.18 | 6.03 ± 1.06 | 1.62 (1.13) | 1.26 (0.99) | 1.47 (1.07) | 1.05 (0.58) |
|  | AA | 24.87 ± 3.43 | 24.08 ± 3.34 | 24.41 ± 3.39 | 9.42 ± 4.53 | 5.20 ± 0.36 | 16.11 ± 6.37 | 5.95 ± 0.93 | 1.56 (1.18) | 1.16 (0.93) | 1.43 (0.96) | 1.01 (0.62) |
| Beta (sem) ^a^ | | -0.14 (0.17) | -0.07 (0.14) | -0.09 (0.11) | 0.16 (0.15) | -0.008 (0.018) | 0.38 (0.27) | -0.014 (0.049) | -0.038 (0.027) | 0.025 (0.024) | -0.001 (0.018) | -0.064 (0.029) |
| *P* value^b^ | | 0.41 | 0.65 | 0.44 | 0.28 | 0.64 | 0.15 | 0.78 | 0.16 | 0.28 | 0.97 | 0.028 |
| rs7961581  *TSPAN8/*  *LGR5* | TT | 25.30 ± 3.45 | 24.15 ± 3.07 | 24.72 ± 3.31 | 8.39 ± 2.95 | 5.22 ± 0.38 | 15.15 ± 5.50 | 5.99 ± 1.05 | 1.65 (1.24) | 1.27 (0.84) | 1.46 (1.08) | 1.09 (0.60) |
|  | TC | 25.16 ± 3.28 | 24.10 ± 3.05 | 24.62 ± 3.21 | 8.30 ± 2.99 | 5.20 ± 0.36 | 14.92 ± 5.00 | 6.03 ± 1.02 | 1.69 (1.10) | 1.18 (0.89) | 1.44 (1.06) | 1.10 (0.59) |
|  | CC | 24.85 ± 3.81 | 24.04 ± 2.81 | 24.49 ± 3.41 | 8.36 ± 3.45 | 5.27 ± 0.37 | 14.85 ± 5.43 | 6.10 ± 1.00 | 1.59 (1.29) | 1.28 (0.77) | 1.36 (0.95) | 1.09 (0.55) |
| Beta (sem) ^a^ | | -0.18 (0.18) | -0.05 (0.17) | -0.11 (0.12) | -0.071 (0.16) | 0.003 (0.20) | -0.18 (0.28) | 0.053 (0.055) | -0.006 (0.028) | -0.021 (0.027) | -0.010 (0.020) | 0.007 (0.032) |
| *P* value^b^ | | 0.33 | 0.77 | 0.40 | 0.65 | 0.90 | 0.51 | 0.34 | 0.83 | 0.43 | 0.63 | 0.83 |
| rs290487  *TCF7L2* | TT | 25.23 ± 3.48 | 24.12 ± 3.00 | 24.68 ± 3.30 | 8.29 ± 3.10 | 5.23 ± 0.37 | 14.90 ± 5.49 | 6.00 ± 1.07 | 1.60 (1.10) | 1.20 (0.78) | 1.37 (0.93) | 1.04 (0.59) |
|  | TC | 25.18 ± 3.43 | 24.16 ± 3.14 | 24.68 ± 3.30 | 8.48 ± 3.03 | 5.20 ± 0.38 | 15.27 ± 5.28 | 6.03 ± 1.03 | 1.68 (1.17) | 1.25 (0.92) | 1.48 (1.18) | 1.12 (0.60) |
|  | CC | 25.51 ± 3.17 | 23.99 ± 2.95 | 24.77 ± 3.15 | 8.26 ± 2.85 | 5.22 ± 0.39 | 14.74 ± 5.17 | 6.06 ± 0.98 | 1.77 (1.33) | 1.18 (0.90) | 1.52 (1.19) | 1.15 (0.59) |
| Beta (sem) ^a^ | | 0.12 (0.15) | -0.04 (0.14) | 0.03 (0.10) | 0.056 (0.13) | -0.009 (0.017) | 0.032 (0.23) | 0.035 (0.045) | 0.046 (0.023) | -0.003 (0.022) | 0.020 (0.017) | 0.011 (0.027) |
| *P* value^b^ | | 0.43 | 0.77 | 0.76 | 0.67 | 0.60 | 0.89 | 0.45 | 0.049 | 0.90 | 0.23 | 0.68 |
| rs12779790  *CDC123/*  *CAMK1D* | AA | 25.12 ± 3.39 | 24.05 ± 2.96 | 24.56 ± 3.22 | 8.51 ± 3.14 | 5.21 ± 0.38 | 15.18 ± 5.32 | 6.03 ± 1.03 | 1.67 (1.20) | 1.23 (0.90) | 1.45 (1.08) | 1.10 (0.61) |
|  | AG | 25.38 ± 3.42 | 24.16 ± 3.07 | 24.75 ± 3.30 | 8.29 ± 3.02 | 5.21 ± 0.38 | 14.91 ± 5.65 | 6.00 ± 1.08 | 1.63 (1.15) | 1.21 (0.86) | 1.42 (1.05) | 1.10 (0.65) |
|  | GG | 25.73 ± 3.92 | 24.63 ± 3.53 | 25.26 ± 3.77 | 7.78 ± 2.32 | 5.28 ± 0.39 | 15.35 ± 4.37 | 5.99 ± 0.95 | 1.65 (0.94) | 1.34 (0.84) | 1.45 (1.03) | 1.00 (0.52) |
| Beta (sem) ^a^ | | 0.25 (0.19) | 0.16 (0.18) | 0.24 (0.13) | -0.26 (0.18) | 0.008 (0.022) | -0.095 (0.31) | -0.029 (0.059) | -0.044 (0.031) | -0.003 (0.029) | -0.021 (0.022) | -0.032 (0.036) |
| *P* value^b^ | | 0.20 | 0.37 | 0.076 | 0.15 | 0.71 | 0.76 | 0.63 | 0.15 | 0.91 | 0.33 | 0.37 |
| rs4607103  *ADAMTS9* | CC | 25.28 ± 3.54 | 24.11 ± 2.92 | 24.70 ± 3.30 | 8.36 ± 2.98 | 5.21 ± 0.39 | 14.98 ± 5.13 | 5.98 ± 1.04 | 1.69 (1.19) | 1.18 (0.81) | 1.44 (1.09) | 1.09 (0.61) |
|  | CT | 25.34 ± 3.37 | 24.15 ± 3.06 | 24.75 ± 3.27 | 8.38 ± 3.04 | 5.22 ± 0.37 | 15.07 ± 5.46 | 6.05 ± 1.04 | 1.62 (1.17) | 1.23 (0.89) | 1.43 (1.03) | 1.13 (0.65) |
|  | TT | 24.76 ± 3.25 | 24.13 ± 3.22 | 24.45 ± 3.24 | 8.41 ± 2.99 | 5.24 ± 0.39 | 15.34 ± 5.53 | 6.11 ± 1.01 | 1.67 (1.16) | 1.29 (0.81) | 1.53 (1.11) | 1.01 (0.46) |
| Beta (sem) ^a^ | | -0.19 (0.15) | 0.01 (0.13) | -0.09 (0.10) | 0.024 (0.13) | 0.014 (0.016) | 0.13 (0.23) | 0.071 (0.044) | 0.019 (0.023) | 0.038 (0.022) | 0.030 (0.016) | 0.008 (0.027) |
| *P* value^b^ | | 0.20 | 0.94 | 0.39 | 0.85 | 0.38 | 0.57 | 0.10 | 0.42 | 0.082 | 0.067 | 0.75 |

Data are expressed as mean ± SD for normally (BMI, FPG and PPG) and median (IQR) for non-normally distributed values (TG and CP_0_)

^a^Calculated using multivariate linear regression analysis adjusted for age, sex and BMI (apart from BMI phenotype)

^b^*P* values are not corrected for multiple testing.

CP_0_, fasting C-peptide; FPG, fasting plasma glucose; PPG, 2h postprandial plasma glucose; TG, triglyceride
